# Preferred and actual place of death in haematological malignancies: a report from the UK haematological malignancy research network

**DOI:** 10.1136/bmjspcare-2019-002097

**Published:** 2020-05-11

**Authors:** Rebecca Sheridan, Eve Roman, Alex G Smith, Andrew Turner, Anne C Garry, Russell Patmore, Martin R Howard, Debra A Howell

**Affiliations:** 1 Epidemiology & Cancer Statistics Group, Department of Health Sciences, University of York, York, North Yorkshire, UK; 2 Faculty of Health and Social Care, Edge Hill University, Ormskirk, Lancashire, UK; 3 Department of Palliative Care, York Hospital, York, YO31 8HE, UK; 4 Queens Centre for Oncology and Haematology, Castle Hill Hospital, Hull, HU16 5JQ, UK; 5 Department of Haematology, York Hospital, York, YO31 8HE, UK

**Keywords:** leukaemia, lymphoma, end of life care, haematological disease, cancer

## Abstract

**Objectives:**

Hospital death is comparatively common in people with haematological cancers, but little is known about patient preferences. This study investigated actual and preferred place of death, concurrence between these and characteristics of preferred place discussions.

**Methods:**

Set within a population-based haematological malignancy patient cohort, adults (≥18 years) diagnosed 2004–2012 who died 2011–2012 were included (n=963). Data were obtained via routine linkages (date, place and cause of death) and abstraction of hospital records (diagnosis, demographics, preferred place discussions). Logistic regression investigated associations between patient and clinical factors and place of death, and factors associated with the likelihood of having a preferred place discussion.

**Results:**

Of 892 patients (92.6%) alive 2 weeks after diagnosis, 58.0% subsequently died in hospital (home, 20.0%; care home, 11.9%; hospice, 10.2%). A preferred place discussion was documented for 453 patients (50.8%). Discussions were more likely in women (p=0.003), those referred to specialist palliative care (p*<*0.001), and where cause of death was haematological cancer (p*<*0.001); and less likely in those living in deprived areas (p=0.005). Patients with a discussion were significantly (p<0.05) less likely to die in hospital. Last recorded preferences were: home (40.6%), hospice (18.1%), hospital (17.7%) and care home (14.1%); two-thirds died in their final preferred place. Multiple discussions occurred for 58.3% of the 453, with preferences varying by proximity to death and participants in the discussion.

**Conclusion:**

Challenges remain in ensuring that patients are supported to have meaningful end-of-life discussions, with healthcare services that are able to respond to changing decisions over time.

## Introduction

Arising in blood and lymph forming tissues, haematological malignancies (leukaemias, lymphomas and myelomas) are collectively the fourth most common cancer among men (after prostate, lung and bowel) and women (after breast, lung and bowel) in the UK and other economically developed countries.^1 2^ With diverse treatments and outcomes, WHO currently recognises more than 100 disease subtypes, some of which are potentially curable but many more that are not.^3^ Chronic myeloproliferative neoplasms (MPN), for example, are considered incurable but symptoms can be treated and the disease normally progresses slowly over time. In contrast, acute myeloid leukaemia (AML) is aggressive and, although potentially curable for some with intensive chemotherapy, it is often rapidly fatal.

Evidence indicates that the end-of-life pathways of patients with haematological malignancies differ from those of people with other cancers. They are, for example, comparatively less likely to receive specialist palliative care (SPC); tend to have fewer, and shorter, hospice stays; are more likely to die in hospital; and are more likely to receive ‘aggressive’ care in their final days.^4–7^ Importantly, these factors are often considered indicators of suboptimal end-of-life care.^8^ There has been increasing research interest in the care pathways and needs of this patient group in recent years,^9^ with closer integration between haematology and palliative care services recommended by the UK Department of Health.^10^ The American Society for Clinical Oncology has also published guidelines recognising that palliative care services should be integrated into routine healthcare for patients with advanced cancer, though no specific recommendation was made regarding haematology.^9^

Dying in a preferred place is generally considered a quality marker for good end-of-life care,^11^ with preference for home death predominating.^12^ However, current evidence suggests that many patients with haematological malignancies do not die in their preferred place.^13^ The present study investigates this issue within the UK’s population-based Haematological Malignancy Research Network (HMRN: www.hmrn.org), which was specifically established to provide information about such subjects.^14^ The aim of the study is threefold: (1) investigate the patient and clinical characteristics associated with actual and preferred place of death in patients with haematological malignancies, (2) determine the concurrence between actual and preferred place of death and (3) characterise discussions in secondary care about preferences including the decisions made, the people involved and changes over time.

## Methods

### Study setting

HMRN was established in 2004, has a catchment of around 4 million people and operates under a legal basis that permits data collection from medical records in 14 hospitals that deliver care in the area, without explicit patient consent. All haematological cancer diagnoses are made and coded by specialist haematopathologists in the Haematological Malignancy Diagnostic Service (HMDS: www.hmds.info), using current classification systems.^3^ Further details of HMRN’s population, structure, ethical approvals, data collection methods and linkages are reported elsewhere.^14^ Briefly, demographic and clinical data are extracted from the hospital records of all patients, and supplemented via routine linkage to national data sources, including the Medical Research Information Service (MRIS), for information on deaths.

### Population

For the present study, all adults (≥18 years) in the HMRN cohort who were newly diagnosed with a haematological malignancy between 1 September 2004 (when HMRN began) and 2012, and who died between 1 September 2011 and 31 August 2012 (to match the most recent death notifications from MRIS at the time of data collection) were eligible for inclusion (n=1041). Seventy-eight (7.5%) patients whose medical records could not be traced by their treating hospital were excluded, many of whom died outside the study area (n=29, 37.2%).

### Study variables

Study variables were selected based on previous literature and insight from clinical members of the steering committee. Routinely collected diagnostic and demographic data (diagnosis, date of diagnosis, age at diagnosis and death, gender and socioeconomic status) were extracted from HMDS and medical records. An area-based measure of the income domain of the Index of Multiple Deprivation was used as the marker of socioeconomic status and was categorised into three distinct groups (1=most affluent; 3=least affluent).^15^ MRIS provided date, place and cause of death; and International Classification of Diseases, 10th Revision codes C91–C96 and D46–D48 indicate death due to haematological malignancy.^16^ Time from diagnosis to death was calculated using the difference between the date of diagnosis and date of death (0–6 months; 6+ months).

Additional data regarding hospital-based outpatient or inpatient discussions about preferred place of death were collected from the hospital records of eligible patients who survived over 14 days after diagnosis. Data were collected by trained study nurses using an extraction form containing the variables of interest, with instructions for completion defined in a detailed protocol. The form and protocol were written by the lead investigator (DAH), piloted by the nurses, amended during working group meetings and finalised by the steering committee. The nurses routinely examined each section of the medical records, including all handwritten notes, typed letters and any other material that might have contained information about advance planning discussions. Forms were checked for quality and consistency at study meetings and during data inputting.

Preferred place of death was grouped into five categories: ‘home’, ‘hospital’, ‘care home’, ‘hospice’ or ‘unknown’; the latter including discussions that did not result in a decision. Discussions were categorised as involving the patient (alone or with relatives) or involving relatives only. The number of discussions recorded per patient was counted and categorised as ‘one’ or ‘more than one’. Where multiple discussions were found, the last recorded discussion before death was used in the analysis. For example, concurrence between actual and preferred place of death was achieved if the patient died in their last recorded preferred place. Information about hospital-based SPC referrals and social circumstances (living alone or not) were also abstracted.

### Statistical analyses

Logistic regression was used to calculate ORs and 95% CIs for associations between patient and clinical factors and (1) place of death (hospital, home, care home, hospice), and (2) the likelihood of a discussion about preferred place of death. Adjustment for age and gender did not alter the results and therefore unadjusted values are reported throughout. Missing data were not imputed; pairwise deletion was used in analyses where appropriate. All analyses were carried out using Stata V.15.1.^17^

### Patient and public involvement

Patients were involved in all aspects of this work including: study design, the funding application, the steering group and dissemination activities.

## Results

A total of 963 patients were included, with 23 haematological malignancy subtypes. As expected, the most common were: diffuse large B-cell lymphoma (16.0%), myeloma (15.9%), AML (12.4%), myelodysplastic syndromes (11.3%), chronic lymphocytic leukaemia (9.4%) and MPN (8.1%).

The majority of patients were male (n=549, 57.0%) and the overall median age at diagnosis was 75.8 years (IQR 66.4–82.9 days; table 1). Patients with MPNs tended to be older at diagnosis and death. Median survival ranged from 3 months (AML) to 39 months (MPN).

**Table 1:** Demographic and clinical characteristics distributed by diagnosis (n=963)

		All patients	DLBCL* n=154 (16.0%)	Myeloma n=153 (15.9%)	AML† n=119 (12.4%)	MDS‡ n=109 (11.3%)	CLL§ n=91 (9.4%)	MPN¶ n=78 (8.1%)	Other n=259 (26.9%)
**Gender**									
Male (%)		549 (57.0)	78 (50.6)	87 (56.9)	66 (55.5)	74 (67.9)	55 (60.4)	39 (50.0)	150 (57.9)
**Age (years, median IQR**)††									
At diagnosis		75.8 (66.4–82.9)	75.0 (64.5–81.5)	72.1 (63.6–80.4)	76.3 (66.2–83.4)	76.1 (68.3–82.2)	76.1 (69.8–84.2)	80.7 (74.8–87.2)	75.9 (66.3–83.4)
At death		77.9 (68.6–85.0)	75.9 (66.3–83.2)	74.6 (66.8–83.3)	77.5 (67.6–83.9)	77.9 (72.2–83.2)	79.5 (73.2–85.9)	83.8 (77.9–89.9)	78.1 (69.3–85.2)
IMD** category (%)									
Most affluent	1	360 (37.4)	59 (38.3)	63 (41.2)	45 (37.8)	44 (40.4)	30 (33.3)	27 (34.6)	92 (35.5)
	2	319 (33.2)	50 (32.5)	50 (32.7)	39 (32.8)	26 (23.9)	30 (33.3)	31 (39.7)	93 (35.9)
Most deprived	3	283 (29.4)	45 (29.2)	40 (26.1)	35 (29.4)	39 (35.8)	30 (33.3)	20 (25.6)	74 (28.6)
**Time from diagnosis to death (%**)									
0–6 months		282 (29.3)	77 (50.0)	40 (26.1)	72 (60.5)	14 (12.8)	14 (15.4)	6 (7.7)	59 (22.8)
6+ months		681 (70.7)	77 (50.0)	113 (73.9)	47 (39.5)	95 (87.2)	77 (84.6)	72 (92.3)	200 (77.2)
Median (IQR)		17.1 (4.4–42.8)	6.2 (1.1–25.8)	25.6 (5.6–42.9)	3.1 (0.9–14.4)	20.4 (8.7–35.4)	36.7 (10.7–52.7)	39.8 (21.3–55.4)	18.0 (7.3–45.4)
**Underlying cause of death**‡‡									
Haematological malignancy (%)		655 (68.0)	117 (76.0)	118 (77.1)	107 (89.9)	73 (67.0)	42 (46.2)	21 (26.9)	177 (68.3)
**Hospital-based SPC referral (n=892)§§**									
Yes (%)		319 (35.8)	50 (38.8)	61 (42.1)	54 (53.5)	29 (27.1)	29 (32.6)	11 (14.1)	85 (35.0)

*Diffuse large B-cell lymphoma.

†Acute myeloid leukaemia.

‡Myelodysplastic syndromes.

§Chronic lymphocytic leukaemia.

¶Myeloproliferative neoplasms.

**Index of Multiple Deprivation (income domain): one patient with missing data.

††IQR.

‡‡Derived from death certificate data, Medical Research Information Service.

§§Specialist palliative care: 71 patients with no data collection due to death within 14 days.

Haematological malignancy was considered the underlying cause of death in 68% of patients (table 1); the other main causes being ischaemic heart diseases (n=45, 4.7%) and chronic lower respiratory diseases (n=33, 3.4%). Approximately one-third (35.8%) of patients were referred to hospital-based SPC services, with a median time from diagnosis to SPC referral of 13.5 months (IQR 2.8–38.9). Patients with AML had the shortest interval (2.8 months, IQR 1.1–10.1) and those with MPN the longest (55 months, IQR 20.2–69.4). Seventy-one patients (7.3%) died within 14 days of diagnosis and were removed from subsequent analyses, as hospital death was considered likely to be unavoidable in this group.

### Place of death

Varying with patient and clinical characteristics, the most common place of death was hospital (n=517, 58%), followed by home (n=178, 20%), care home (n=106, 11.9%) and hospice (n=91, 10.2%). Table 2 presents the demographic and clinical characteristics of patients distributed by actual place of death.

**Table 2:** Demographic and clinical characteristics distributed by actual place of death (n=892)

		All deaths	Actual place of death			
			Hospital n = 517 (58.0%)	Home n = 178 (20.0%)	Care home n = 106 (11.9%)	Hospice n = 91 (10.2%)
**Gender**						
Male (%)		520 (58.3)	319 (61.7)	108 (60.7)	42 (39.6)	51 (56.0)
**Age at death (years, %)**						
≤74		363 (40.7)	231 (44.7)	80 (44.9)	7 (6.6)	45 (49.5)
75+		529 (59.3)	286 (55.3)	98 (55.1)	99 (93.4)	46 (50.5)
IMD category* (%)						
Most affluent	1	328 (36.8)	196 (38.0)	63 (35.4)	35 (33.0)	34 (37.4)
	2	303 (34.0)	165 (32.0)	64 (36.0)	44 (41.5)	30 (33.0)
Most deprived	3	260 (29.2)	155 (30.0)	51 (28.7)	27 (25.5)	27 (29.7)
**First diagnosis (%)**						
DLBCL†		129 (14.5)	71 (13.7)	23 (12.9)	22 (20.8)	13 (14.3)
Myeloma		145 (16.3)	90 (17.4)	35 (19.7)	11 (10.4)	9 (9.9)
AML‡		101 (11.3)	61 (11.8)	22 (12.4)	7 (6.6)	11 (12.1)
MDS§		107 (12.0)	69 (13.3)	22 (12.4)	6 (5.7)	10 (11.0)
CLL¶		89 (10.0)	49 (9.5)	18 (10.1)	12 (11.3)	10 (11.0)
MPN**		79 (8.7)	34 (6.6)	20 (11.2)	18 (17.0)	6 (6.6)
Other		243 (27.2)	143 (27.7)	38 (21.3)	30 (28.3)	32 (35.2)
**Time from diagnosis to death (%)**						
0–6 months		211 (23.7)	138 (26.7)	34 (19.1)	27 (25.5)	12 (13.2)
6+ months		681 (76.3)	379 (73.3)	144 (80.9)	79 (74.5)	79 (86.8)
**Underlying cause of death††**						
Haematological malignancy (%)		602 (67.5)	353 (68.3)	117 (65.7)	64 (60.4)	68 (74.7)
**Patient lived alone**						
Yes (%)		247 (27.7)	144 (27.9)	45 (25.3)	22 (20.8)	36 (39.6)
**Hospital-based SPC‡‡ referral**						
Yes (%)		319 (35.8)	167 (32.3)	66 (37.1)	19 (17.9)	67 (73.6)
**Preferred place of death discussion recorded**						
Yes (%)		453 (50.8)	217 (42.0)	103 (57.9)	61 (57.5)	72 (79.1)

*Index of Multiple Deprivation (income domain): one patient with missing data.

†Diffuse large B-cell lymphoma.

‡Acute myeloid leukaemia.

§Myelodysplastic syndromes.

¶Chronic lymphocytic leukaemia.

**Myeloproliferative neoplasms.

††Derived from death certificate data, Medical Research Information Service.

‡‡Specialist palliative care.

A number of factors were significantly associated with place of death (table 3). As expected, compared with those dying in hospital, people dying in a care home were more likely to be older (OR 11.42, 95% CI 5.21 to 25.10) and female (OR 2.46, 95% CI 1.60 to 3.77). In common with patients dying at home, they were also more likely to have an MPN, the most indolent of included diagnoses and typically managed in the home setting; the corresponding ORs were 2.16 (95% CI 1.04 to 4.49) and 2.30 (95% CI 1.13 to 4.69) for care home and home, respectively. As expected, patients who died closer to diagnosis were less likely to die at home (OR 0.65, 95% CI 0.43 to 0.99) or in a hospice (OR 0.42, 95% CI 0.22 to 0.79); with those dying in a hospice being more likely to have lived alone (OR 1.70, 95% CI 1.07 to 2.69) and to have had an SPC referral (OR 5.85, 95% CI 3.54 to 9.66). Across all comparisons, patients who had a preferred place of death discussion recorded in their medical notes were more likely to die in a non-hospital setting (eg, hospital vs hospice: OR 5.24, 95% CI 3.07 to 8.94).

**Table 3:** OR and 95% CI of patient demographic and clinical characteristics associated with actual place of death*

		Home		Care home		Hospice	
		OR (95% CI)	P value	OR (95% CI)	P value	OR (95% CI)	P value
**Gender**							
Male		1.00	–	1.00	–	1.00	–
Female		1.04 (0.74 to 1.48)	0.808	2.46 (1.60 to 3.77)	**<0.001**	1.26 (0.81 to 1.98)	0.309
**Age at death (years**)							
≤74		1.00	–	1.00	–	1.00	–
75+		0.99 (0.70 to 1.39)	0.951	11.42 (5.21 to 25.10)	**<0.001**	0.83 (0.53 to 1.29)	0.400
IMD category*							
Most affluent	1	1.00	–	1.00	–	1.00	–
	2	1.21 (0.81 to 1.81)	0.363	1.49 (0.92 to 2.44)	0.109	1.05 (0.62 to 1.79)	0.863
Most deprived	3	1.02 (0.67 to 1.57)	0.914	0.98 (0.57 to 1.68)	0.929	1.00 (0.58 to 1.74)	0.988
**First diagnosis**							
DLBCL†		1.00	–	1.00	–	1.00	–
Myeloma‡		1.52 (0.85 to 2.75)	0.159	0.47 (0.22 to 1.03)	0.058	0.76 (0.32 to 1.82)	0.540
AML§		1.10 (0.57 to 2.09)	0.794	0.36 (0.15 to 0.89)	**0.027**	1.01 (0.43 to 2.37)	0.988
MDS¶		1.23 (0.64 to 2.37)	0.535	0.41 (0.17 to 1.01)	0.052	1.03 (0.43 to 2.49)	0.942
CLL**		1.38 (0.69 to 2.78)	0.364	0.96 (0.44 to 2.09)	0.921	1.42 (0.58 to 3.46)	0.443
MPN††		2.30 (1.13 to 4.69)	**0.021**	2.16 (1.04 to 4.49)	**0.038**	1.28 (0.45 to 3.62)	0.647
Other		0.97 (0.55 to 1.72)	0.925	0.81 (0.44 to 1.47)	0.481	1.47 (0.74 to 2.95)	0.273
**Time from diagnosis to death (months**)							
0–6 months		0.65 (0.43 to 0.99)	**0.044**	0.94 (0.58 to 1.51)	0.795	0.42 (0.22 to 0.79)	**0.007**
6+ months		1.00	–	1.00	–	1.00	–
**Underlying cause of death‡‡**							
Haematological malignancy		0.89 (0.62 to 1.28)	0.531	0.71 (0.46 to 1.09)	0.116	1.37 (0.83 to 2.28)	0.220
Other		1.00	–	1.00	–	1.00	–
**Patient lived alone§§**							
Yes		0.88 (0.59 to 1.29)	0.506	0.68 (0.41 to 1.13)	0.134	1.70 (1.07 to 2.69)	**0.025**
No/unknown		1.00	–	1.00	–	1.00	–
**Hospital-based SPC¶¶ referral**							
Yes		1.24 (0.87 to 1.72)	0.245	0.46 (0.27 to 0.78)	**0.004**	5.85 (3.54 to 9.66)	**<0.001**
No		1.00	–	1.00	–	1.00	–
**Preferred place of death discussion recorded**							
Yes		1.90 (1.34 to 2.68)	**<0.001**	1.87 (1.34 to 2.68)	**0.005**	5.24 (3.07 to 8.94)	**<0.001**
No		1.00	–	1.00	–	1.00	–

Bold font indicates statistical significance.

*Reference category is hospital death.

†Index of Multiple Deprivation (income domain): one patient with missing data.

‡Diffuse large B-cell lymphoma.

§Acute myeloid leukaemia.

¶Myelodysplastic syndromes.

**Chronic lymphocytic leukaemia.

††Myeloproliferative neoplasms.

‡‡Derived from death certificate data, Medical Research Information Service.

§§Patient lived alone: ‘No’ includes 24 patients where it was ‘unknown’.

¶¶Specialist palliative care: ‘No’ includes 25 patients where SPC is ‘Unknown’.

### Preferred place of death discussions

Discussion(s) about preferred place of death were documented in the medical records of 50.8% of patients (table 4). Patients who had a preferred place discussion were more likely to be female (OR 1.51, 95% CI 1.15 to 1.97, p*=*0.003), while patients living in the most deprived areas were less likely to have had a discussion (OR vs least deprived 0.69, 95% CI 0.50 to 0.96, p=0.005). A smaller proportion of these patients died of their haematological malignancy (most deprived, 63.9%; least deprived, 71.3%) or had evidence of SPC referral (most deprived, 27.3%; least deprived, 44.8%). Notably, both of these factors were significantly associated with increased likelihood of having a preferred place discussion (haematological cause of death: OR 2.52, 95% CI 1.89 to 3.37, p<0.001; SPC referral: OR 8.93, 95% CI 6.40 to 12.46, p<0.001). Age at death, time between diagnosis and death, and the patient’s social circumstances had no significant impact on whether a discussion was recorded.

**Table 4:** Likelihood of patients having a preferred place of death discussion by demographic and clinical characteristics

Factors		Preferred place discussion recorded		OR (95% CI)
		Yes n=453 (50.8%)	No n=439 (49.2%)	
**Gender**				
Male		242 (46.5)	278 (53.5)	1.00
Female		211 (56.7)	161 (43.3)	**1.51 (1.15 to 1.97**)
**Age at death (years**)				
≤74		192 (52.9)	171 (47.1)	1.15 (0.88 to 1.51)
75+		261 (49.3)	268 (50.7)	1.00
IMD category*				
Most affluent	1	189 (57.6)	139 (42.4)	1.00
	2	137 (45.2)	166 (54.8)	**0.61 (0.44 to 0.83**)
Most deprived	3	126 (48.5)	134 (51.5)	**0.69 (0.50 to 0.96**)
**Time from diagnosis to death (months**)				
0–6 months		113 (53.6)	98 (46.4)	1.16 (0.85 to 1.58)
6+months		340 (49.9)	341 (50.1)	1.00
**Underlying cause of death†**				
Haematological malignancy		350 (58.1)	252 (41.9)	**2.52 (1.89 to 3.37**)
Other		103 (35.5)	187 (64.5)	1.00
**Patient lived alone‡**				
Yes		138 (55.9)	109 (44.1)	1.33 (0.99 to 1.78)
No		315 (48.8)	330 (51.2)	1.00
**Hospital-based SPC referral§**				
Yes		261 (81.8)	58 (18.2)	**8.93 (6.40 to 12.46**)
No		192 (33.5)	381 (66.5)	1.00

Bold font indicates statistical significance.

*Index of Multiple Deprivation (income domain): one patient with missing data.

†Derived from death certificate data, Medical Research Information Service.

‡No includes 24 where patient living circumstances were ‘unknown’.

§Specialist palliative care: ‘No’ includes 25 patients where SPC is ‘Unknown’.

The most common last recorded preference was for home death (n=184, 40.6%), followed by hospice (n=82, 18.1%), hospital (n=80, 17.7%) and care home (n=64, 14.1%). Not including those with an unknown preference, 271 (66.1%) patients died in their final preferred place (table 5). Almost all those with hospital listed as the preferred place died in hospital (n=77, 96.3%) while roughly half wanting to be at home died at home (n=95, 51.6%). When patients did not die in their preferred place, or had an unknown preference, the most common place of death was hospital. The majority of last recorded discussions involved patients (n=323, 71.6%); this was most common when the preferred place was home (87%) and least common when the preferred place was hospital (48.1%). Proximity to death was also important; when hospital was preferred, 85% of discussions were held within 7 days of death reducing to 50%, 35.9% and 25% if it was hospice, home or care home, respectively. Overall, median time from last discussion to death was 8 days (IQR 3–29).

**Table 5:** Actual place of death, patient involvement and number of discussions by the last recorded preferred place, n=453 with discussion recorded

	All deaths	Preferred place at discussion closest to death (%)				
		Hospital n=80 (17.7%)	Home n=184 (40.6%)	Care home n=64 (14.1%)	Hospice n=82 (18.1%)	Unknown n=43 (9.5%)
**Actual place of death**						
Hospital	217 (47.9)	77 (96.3)	66 (35.9)	15 (23.4)	28 (34.1)	31 (72.1)
Home	103 (22.7)	2 (2.5)	95 (51.6)	–	1 (1.2)	5 (11.6)
Care home	61 (13.5)	1 (1.3)	6 (3.3)	48 (75.0)	2 (2.4)	4 (9.3)
Hospice	72 (15.9)	–	17 (9.2)	1 (1.6)	51 (62.2)	3 (7.0)
Involved in discussion (n=451)*						
Patient (alone/with relative)	323 (71.6)	38 (48.1)	160 (87.0)	32 (50.0)	65 (80.2)	28 (65.1)
Relative(s) only	122 (28.4)	41 (51.9)	24 (13.0)	32 (50.0)	16 (19.8)	15 (34.9)
**Number of discussions**						
One	189 (41.7)	24 (30.0)	92 (50.0)	32 (50.0)	24 (29.3)	17 (39.5)
More than one	264 (58.3)	56 (70.0)	92 (50.0)	32 (50.0)	58 (70.7)	26 (60.5)
No change in preference	118 (44.7)	8 (14.3)	74 (80.4)	13 (40.6)	13 (22.4)	10 (38.5)

*Not including those whose involvement was ‘Unknown’ (n=2).

Over half of those with a preferred place discussion (n=264, 58.3%) had more than one discussion (range 1–7), with variation seen by last recorded preference (hospice, 70.7%; hospital, 70.0%; unknown, 60.5%; care home, 50.0%; home, 50.0%). While 118 (44.7%) stated the same preference at their first and last recorded discussion, 146 (55.3%) changed (table 5). For patients whose last recorded preference was hospital, 14.3% had the same initial preference. When home was the last choice, 80.4% also chose home at first discussion.

## Discussion

### Principal findings

The most common place of death was hospital, with the lowest frequency seen in those with more indolent disease subtypes. Half of all patients in the study had a recorded preferred place of death discussion. This was significantly more likely for women, people with SPC referrals and those dying from their haematological cancer, and less likely in patients living in deprived areas. As expected, the most common preference was for home death, especially at first discussion; although one in five final discussions resulted in preference for hospital death. Two-thirds died in their preferred place including most of those preferring hospital and around half wanting to die at home. Having a discussion significantly increased the likelihood of non-hospital death. Most patients had multiple discussions, clearly demonstrating an ongoing, dynamic process, with decisions determined by who was involved in the conversation (patients and/or relatives) and proximity to death.

### Strengths and limitations

This is the largest study we are aware of to examine preferred and actual place of death, and the characteristics of associated discussions in patients with haematological malignancies. It is also the only work examining decisions made in this context by relatives alone. Setting the study within a well-established population-based cohort and hand searching secondary care records (electronic and paper) for information ensured data collection was thorough and all relevant discussions and decisions were identified. We intentionally included all patients newly diagnosed with haematological malignancies between 2004 and 2012, who died during a 1-year period. This ensured variable follow-up time (maximum 7 years; minimum of 2 weeks) and inclusion of patients with differing experiences/survival outcomes, thereby facilitating examination of issues such as how place of death is impacted by survival duration, and time from SPC referral to death. Including all 2011–2012 deaths ensured findings reflected contemporary end-of-life clinical practice. As all adult deaths (≥18 years, regardless of gender, deprivation and diagnosis) were included, our study is likely to be generalisable to other locations with similar healthcare systems and settings.

Data collection relied on information written in medical records by clinical staff, which did not always specify the exact purpose of discussions. Unclear documentation was examined during study meetings and included if it was agreed that the excerpt related to place of care or place of death. These are clearly different issues,^18^ however, and improvements in the documentation of advance care planning may mean it is possible to distinguish between these issues in the future. Due to difficulties accessing data from multiple settings, we focused on hospital activities. Consequently, data regarding discussions and SPC referrals in primary and community care are not included. While data collection took place several years ago, our findings are consistent with more recent data,^19^ and preferred and actual place of death remains an important issue in both research and practice, alongside advance planning more generally (eg, http://respectprocess.org.uk).

### Comparison with existing literature

Two previous studies involving patients with haematological malignancies have observed higher proportions of preferred place discussions than the current study,^11 20^ possibly due to these projects including only patients known to SPC services—the association between SPC referral and likelihood of a discussion being clearly highlighted in the current study, and other work.^13^ Studies examining likelihood of a discussion by gender also found women more likely to have discussed preferences,^13^ with men said to be more reluctant to discuss their impending death, even after interventions to redress this imbalance.^21^

Although we could not find evidence to corroborate the relationship between increasing deprivation and decreased preference discussions in the haematology context, studies examining related issues provide compelling evidence of other disadvantages, including fewer SPC referrals, more hospital deaths (though not statistically significant in the present study), and indeed, poorer quality care.^19 22^ Across all included patients in our study, we found those living in increasingly deprived areas were comparatively more likely to die from comorbidities rather than their haematological malignancy. The existence of such comorbidities may lead to patients being managed by multiple specialities,^13^ thus possibly obscuring responsibility for instigating end-of-life conversations and resulting in missed opportunities.

The characteristics we noted about discussions (recurrent conversations, changing preferences and disparity between patient and relatives’ views) have been reported in other studies.^13 18 23 24^ As expected, preferences generally shifted from death at home towards hospital death.^13 19^ A comprehensive systematic review reported that 20% of patients changed their preferences (range 2%–80%).^25^ In our study population, we observed a higher proportion of change (55.3%), which may be due to circumstance as much as choice. For example, uncertain disease trajectories and prognoses are common in haematological malignancies and may cause sudden deterioration and death, leaving little time to organise transfer home, or to a hospice, to die.^24 26–28^ Alternatively, the inability of patients or family/carers to cope at home may mean hospital becomes the preferred, or *de facto*, place of death, even when home was preferred and planned.^24 29^ This may also explain some of the observed discrepancies between patient and family members’ preferences.

Our observed concurrence between preferred and actual place of death (66.1%) is similar to earlier research in the same area (63.4%),^13^ and well within the range of studies included in a systematic review (30%–89%),^30^ although the latter incorporated both cancer and non-cancer studies and noted difficulties comparing work due to varied settings, populations and methods. Others have also reported large numbers of hospital deaths among patients with haematological malignancies,^6^ and described indecision and unknown preferences, either because patients had not been asked, were unwilling to have a discussion, or had no preference.^23 26^ Research described those without a discussion dying in hospital more often,^11 13 20^ with those surviving longer being more likely to die at home or in hospice,^31^ probably because there was more time to arrange this.^27^

### Implications for practice and future research

Having a preferred place of death discussion, and the decision documented, is important if patient preferences at the end-of-life are to be achieved.^11 13 20^ Ensuring all patients have the opportunity to discuss this in a supportive, pragmatic and compassionate way is therefore paramount. This could be facilitated by strengthened training for medical professionals to improve their ability and confidence in end-of-life discussions.^32^ The increased likelihood of preference discussions, and of death in hospice in those with SPC input suggests further haematology/SPC integration can also be beneficial. Greater support may also be required for haematology staff providing end-of-life care and advance planning; while some consider this a necessary skill and a rewarding experience,^27 28 33^ issues are reported with providing adequate emotional and psychological support for patients and families on busy acute wards.^28^ Regarding documentation of discussions and decisions, the introduction of the Electronic Palliative Care Coordinating Systems (EPaCCS) in England may facilitate recording between care settings, though challenges implementing EPaCCS remain.^34^

Despite some changes in place of death over time, hospital death will likely predominate into the future.^35^ It is therefore vital that measures are put in place in this setting to improve the experiences of patients and their families at this time.^36^ While choice in dying remains important, home death is increasingly considered to have become ‘a quality marker in itself’, rather than a true, adequately resourced choice that can meet changing needs.^37^ Indeed, some consider end-of-life hospitalisation to be unavoidable and justified^38^; with the delivery of 24 hours quality care by experts, being pain/symptom free, having family present and not being a burden said to have greater significance.^36 39^ Importantly, where preferences are not met and hospital-death occurs, bereaved relatives often later describe hospital as the ‘right’ place.^19 24^ Findings such as these bring into question the suitability of concurrence between preferred and actual place of death as an indicator of quality^36 40^ Further research is required into the impact of deprivation and other socioeconomic factors on preferences, reasons for not discussing place of death, not stating a preference or changing preference. More also needs to be known about the role of haematologists and specialist nurses in discussing end-of-life preferences, to understand if and how gaps in advance planning could be addressed.
